# Physicochemical, Morphological, and Functional Characterization of Edible Anthocyanin-Enriched *Aloe* *vera* Coatings on Fresh Figs (*Ficus carica* L.)

**DOI:** 10.3390/gels8100645

**Published:** 2022-10-11

**Authors:** Sawsan Ali Al-Hilifi, Rawdah Mahmood Al-Ali, Orass T. Al-Ibresam, Nishant Kumar, Saeed Paidari, Anka Trajkovska Petkoska, Vipul Agarwal

**Affiliations:** 1 Department of Food Science, College of Agriculture, University of Basrah, Basrah 61004, Iraq; 2 Department of Food Science and Technology, National Institute of Food Technology Entrepreneurship and Management, Sonepat 131028, India; 3 Department of Food Science and Technology, Isfahan (Khorasgan) Branch, Islamic Azad University, Isfahan 81551-39998, Iran; 4 Faculty of Technology and Technical Social Sciences, St. Kliment Ohridski University-Bitola, Dimitar Vlahov, 1400 Veles, North Macedonia; 5 Cluster for Advanced Macromolecular Design (CAMD), School of Chemical Engineering, University of New South Wales, Sydney, NSW 2052, Australia

**Keywords:** coating, anthocyanin, fruit decay, postharvest shelf life, *Aloe vera*, antioxidant, fig

## Abstract

In the present investigation, *Aloe vera* gel (AVG)-based edible coatings enriched with anthocyanin were prepared. We investigated the effect of different formulations of aloe-vera-based edible coatings, such as neat AVG (T1), AVG with glycerol (T2), *Aloe vera* with 0.2% anthocyanin + glycerol (T3), and AVG with 0.5% anthocyanin + glycerol (T4), on the postharvest quality of fig (*Ficus carica* L.) fruits under refrigerated conditions (4 °C) for up to 12 days of storage with 2-day examination intervals. The results of the present study revealed that the T4 treatment was the most effective for reducing the weight loss in fig fruits throughout the storage period (~4%), followed by T3, T2, and T1. The minimum weight loss after 12 days of storage (3.76%) was recorded for the T4 treatment, followed by T3 (4.34%), which was significantly higher than that of uncoated fruit (~11%). The best quality attributes, such as the total soluble solids (TSS), titratable acidity (TA), and pH, were also demonstrated by the T3 and T4 treatments. The T4 coating caused a marginal change of 0.16 in the fruit titratable acidity, compared to the change of 0.33 in the untreated fruit control after 12 days of storage at 4 °C. Similarly, the total soluble solids in the T4-coated fruits increased marginally (0.43 °Brix) compared to the uncoated control fruits (>2 °Brix) after 12 days of storage at 4 °C. The results revealed that the incorporation of anthocyanin content into AVG is a promising technology for the development of active edible coatings to extend the shelf life of fig fruits.

## 1. Introduction

In the past decade, there has been remarkable growth in the development of bio-based and active edible packaging to ensure food safety and quality and reduce postharvest losses of fruits and vegetables [1,2,3,4,5]. Edible packaging is an ecofriendly and sustainable approach to maintaining the quality attributes of fruits and vegetables during storage by (i) minimizing lipid peroxidation, (ii) altering the respiration rate, (iii) reducing weight loss, and (iv) maintaining other quality attributes [6,7,8]. Furthermore, edible coatings also increase fruit microbiological safety and protect them from the effects of external environmental conditions, hence extending their shelf lives [9]. Moreover, the application of functional agents/compounds such as antioxidants, antimicrobials, and nutraceuticals in edible coatings improves the quality attributes and postharvest characteristics of fruits and vegetables [10,11,12,13]. 

Figs (*Ficus carica.* L.) are a popular fruit due their health benefits and functional properties, including boosting immunity and being rich in fiber and antioxidant compounds. However, they are highly perishable due to their susceptibility to deterioration, oxidation, and microbial growth [14,15,16,17,18]. These fruits are also sensitive to microbial contamination under cold storage conditions, resulting in an unpleasant taste and aroma that affect consumer acceptability [19]. The postharvest shelf life of fig fruits can be extended using various technologies, i.e., cold storage, modified atmosphere packaging, controlled atmospheres, vacuum application, and edible packaging (coating) [20,21,22,23].

Edible coating is an alternative postharvest management strategy to extend the shelf life of fig fruits by preserving their quality attributes [17,24]. Different types of edible coatings based on polysaccharides, proteins, lipids, or composites have been used for fruit and vegetable preservation. The advantages of these edible coatings include their eco-friendliness, biodegradability in nature, and ability to extend the shelf life of fruits and vegetables [6,7]. *Aloe vera* is a succulent plant of the *Asphodelaceae* family of the genus Aloe that has been used as a medicinal plant for millennia. *Aloe vera* gel (AVG) is the pulp produced by *Aloe vera* plants, and its gelatinous matrix can be used to produce natural edible coatings. Its applicability in the preservation of certain fruits has been reported previously [25]. The preservation effectiveness of AVG coatings has been attributed to its ability to (i) reduce phenolic oxidation, (ii) inhibit peroxidase and polyphenol oxidase enzyme activity, and (iii) reduce the browning of fruits while preserving their quality from the detrimental effects of weight loss, enzymatic browning, electrolytic leakage, respiration rate degradation, and chlorophyll degradation [26]. Fruit preservation quality can be assessed in terms of firmness, visual appeal, nutrient content, and freshness [27,28]. AVG is considered an excellent source of nutritional and phytochemical compounds such phenols, flavonoids, terpenoids, lectins, and fatty acids. AVG also contains vitamins and polysaccharides, which function as natural antioxidants [29]. AVG has shown antiviral, antibacterial, laxative, antioxidant, anti-inflammation, anticancer, antidiabetic, antiallergic, immunostimulatory, and UV-protective properties due to presence of high amounts of bioactive compounds [30]. However, neat AVG is highly hydrophilic, which limits its functional duration as a fruit protective coating. To this end, AVG is supplemented with other constituents to improve its properties and longevity. While a range of different coatings have been developed and tested on various fruits [31,32], to the best of our knowledge, no study has explored the potential of anthocyanin (as a natural antioxidant) and AVG supplemented with anthocyanin in natural edible coatings.

The present study aimed to investigate the physicochemical, morphological, and functional characteristics of AVG-based edible coatings enriched with anthocyanin extracted from onion peel. We assessed the effects of different treatments based on the developed AVG edible coating with or without anthocyanin on the shelf life of *Ficus carica* fruit during 12 days of storage at 4 °C.

## 2. Results and Discussion 

### 2.1. Physicochemical Properties of Aloe vera Gel (AVG)

Table 1 depicts the physicochemical observations for the AVG. The results showed that the AVG had a moisture content of 97.52%, a pH of 5.29 with 0.06% acidity, a carbohydrate content of 0.65%, a viscosity of 4.66, a refractive index of 1.33, and TSS of 3.10 °Bx. Our results were in agreement with previous studies [33,34,35]. The obtained refractive index of 1.33 was in line with the International Aloe Science Council’s recommendation (IASC). A gel’s refractive index is a physical measure that defines its purity when compared to double-distilled water. The optimum treatment for the coating process is a gel with the lowest refractive index. Impurities in the extracted gel are indicated by a higher refractive index [36].

### 2.2. Microstructure Analysis

**Figure 1:**
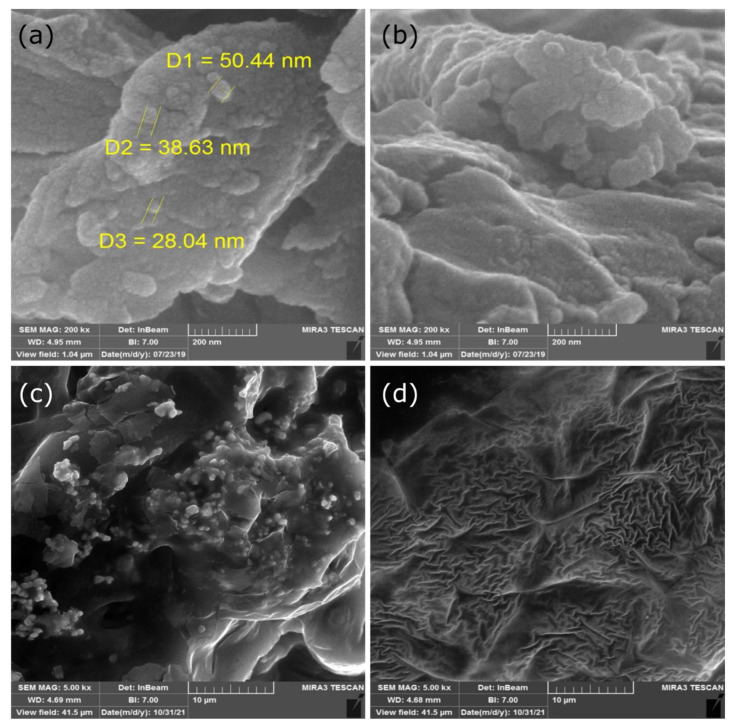
SEM images showing the microstructure of AVG-based edible coatings. (**a**) AVG (T1), (**b**) AVG + glycerol (T2), (**c**) AVG + glycerol + anthocyanin + 0.2% (T3), (**d**) AVG + glycerol + anthocyanin + 0.5% (T4). Scale bar: (**a**,**b**) = 200 nm, (**c**,**d**) = 10 µm. Magnifications: (**a**,**b**) 200,000×, (**b**,**c**) 5000×.

The microstructure analysis was conducted using SEM (Figure 1). The AVG image showed a more organized and smoother structure compared to the others, with an intact parenchymal cell of a well-rounded shape and characteristic diameter, whose dimensions were in the range of 28–50 nm due to the presence of a high water content. Similarly, the close contact between the walls of adjacent cells was in line with previous reports [37,38]. Compared to the control samples, all coating samples exhibited decreased intracellular integrity and cell shape regularity. The results showed that the incorporation of glycerol and anthocyanin into the AVG introduced ruptured cell structures. The microstructure of the AVG with anthocyanin content was more shrinkable and less porous compared to the others. The pore sizes of the AVG + glycerol + anthocyanin coating (T3 and T4) samples (Figure 1c,d) were smaller than those of the neat AVG and AVG + glycerol coatings (Figure 1a,b). The observed differences were attributed to the weaker hydrogen bonds between the carboxylic group of the AVG and anthocyanin. An increased concentration of anthocyanin in the AVG resulted in a more complex and rougher film microstructure. The obtained results were similar to those of a previous report on aloe-vera–gelatin–glycerol edible films enriched with *Pimenta dioica* L. Merrill essential oil [39]. In this study, it was reported that the incorporation of the active ingredient (*Pimenta dioica* L. Merrill essential oil) affected the microstructure of the AV–gelatin–glycerol-based films by increasing the roughness and the flocculation rate on the surface.

### 2.3. Effect of AVG-Based Edible Coatings on Postharvest Quality of Fig Fruits

#### 2.3.1. Weight Loss

**Figure 2:**
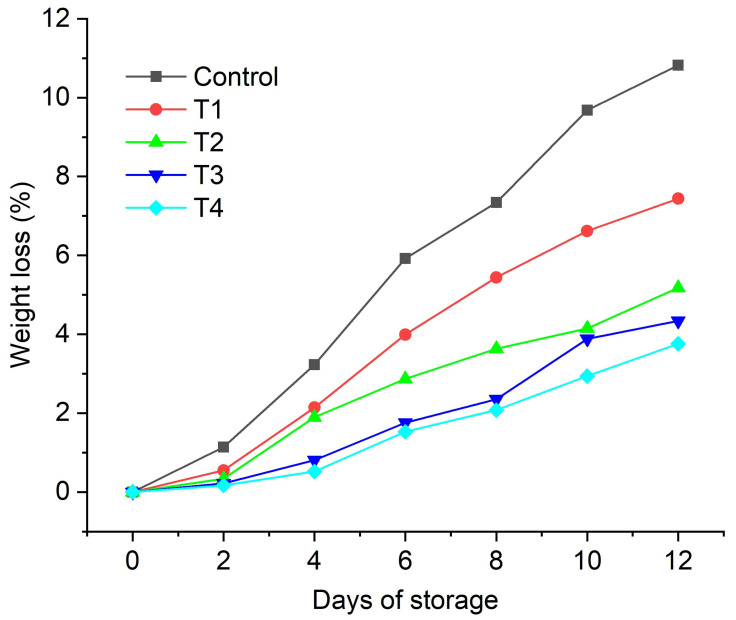
Effect of edible coatings on weight loss of *Ficus carica* fruits: uncoated control, AVG (T1), AVG + glycerol (T2), AVG + glycerol + anthocyanin + 0.2% (T3), AVG + glycerol + anthocyanin + 0.5% (T4). Data are presented as mean ± SD, (*n* = 3) (error bars are significantly smaller than the data points).

Figs are highly susceptible to weight loss due to their thin peels, which allow for rapid water loss and tissue deterioration. Figure 2 presents the weight loss (%) of the coated and uncoated figs. We observed significantly reduced weight loss (*p* < 0.05) in the coated fruits after 10 days of storage (<2.94%) compared to the uncoated figs (maximum weight loss of 9.68%). The combination of anthocyanin with AVG + glycerol effectively contributed to reducing weight loss. The observed reduction in weight loss in the coated fruits was attributed to the formation of a semi-permeable barrier that prevented water loss [25,40]. Furthermore, the superior performance of the AVG + glycerol + anthocyanin coating could have been caused by a reduction in the water loss of the fruits due the crosslinking of anthocyanin, glycerol, and AVG [41,42]. In the presence of plant extracts (AVG), the properties of biopolymeric films can be changed as a result of interactions between the biopolymer and polyphenolic compounds (anthocyanin) [43]. The weight loss measured for the fruits coated using the developed (AVG + glycerol-based) edible coatings was either similar to or significantly better than the results obtained by previous studies [17,44,45]. For example, contrary to our study, the application of zein containing cystein (0.2%), ascorbic acid (0.2%), and jamun leaf extract (0.2%) coatings on jamun fruit resulted in significantly higher weight loss as the storage period progressed [46]. In another study, an *Aloe vera* and gum tragacanth coating applied to button mushrooms was shown to cause a significant weight loss of ~40–50% over the 13-day storage period, compared to the figure of ~2–5% obtained for our AVG + glycerol + anthocyanin-coated figs over the 12-day storage period [47]. The difference between the abovementioned work [47] and our study could be attributed to a combination of (i) the superior performance of our coatings and (ii) the higher water content of button mushrooms compared to fig fruits. Furthermore, the significant difference in weight loss between the uncoated and coated fruits observed in our study was in disagreement with a previous report on the application of quinoa protein/chitosan coatings containing a thymol nanoemulsion on refrigerated strawberries, which showed no difference between the coated and uncoated fruit [48]. The observed disagreement could be attributed to the significant differences in the (fruit) surface adhesion of the two coatings, i.e., chitosan + quinoa protein + thymol nanoemulsion and AVG + glycerol + anthocyanin.

#### 2.3.2. pH

**Figure 3:**
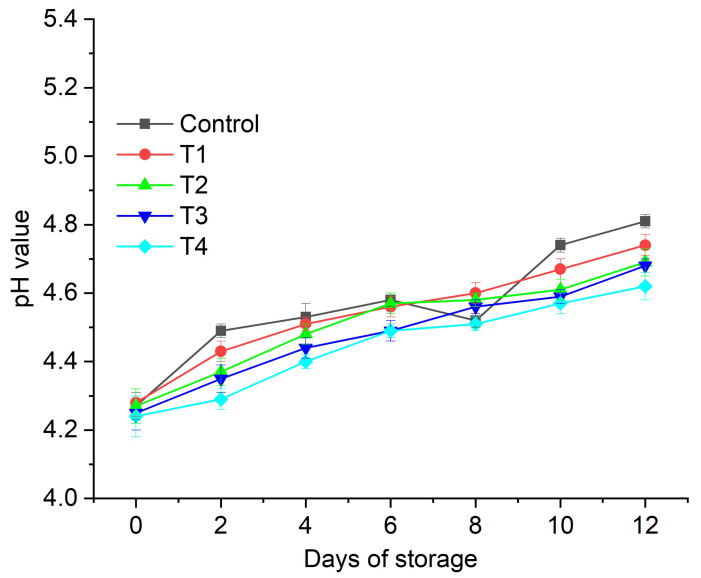
Effect of edible coatings on pH of *Ficus carica* fruits: uncoated control, AVG (T1), AVG + glycerol (T2), AVG + glycerol + anthocyanin + 0.2% (T3), and AVG + glycerol + anthocyanin + 0.5% (T4). Data are presented as mean ± SD, (*n* = 3).

The change in pH is an important indicator of fruit properties, with an increase in pH indicating the ripening and oxidation of the fruit over time. To prolong a fruit’s shelf life, it is important that the change in pH is marginal over the storage period. The pH analysis of the juice extracted from the coated and uncoated fruits revealed a gradual increase as the storage period progressed (Figure 3). Compared to the control fruits (T0—water-washed fruits), the coated fruits exhibited a lower increase in pH values over time. We observed a maximum increase in pH for the control fruits (from ~4.3 at day 0 to ~4.7 at day 12) as the storage period progressed. The lowest pH increase was observed for the figs coated with T4 (AVG + glycerol + anthocyanin 0.5%) over the storage period. Coatings are known to reduce the respiratory and metabolic rates of fruit, thereby limiting the utilization of organic acids and restricting the pH change over the storage period [49]. The addition of active compounds such as anthocyanin promotes coatings’ functional performance, enhancing the stability; quality (reducing biochemical deterioration, enzymatic browning, and the development of off-flavors); and safety of foods [50,51]. Similar results in terms of marginal changes in pH value have been reported previously for an AVG-based edible coating on freshly cut papaya [28], a nanostructured lipid carriers + cinnamon essential oil coating on tangerine [52], and a pectin + candelilla wax + aloe mucilage + glycerol + polyphenol Larrea leaf extract coating on avocados [53].

#### 2.3.3. Titratable Acidity (TA)

**Figure 4:**
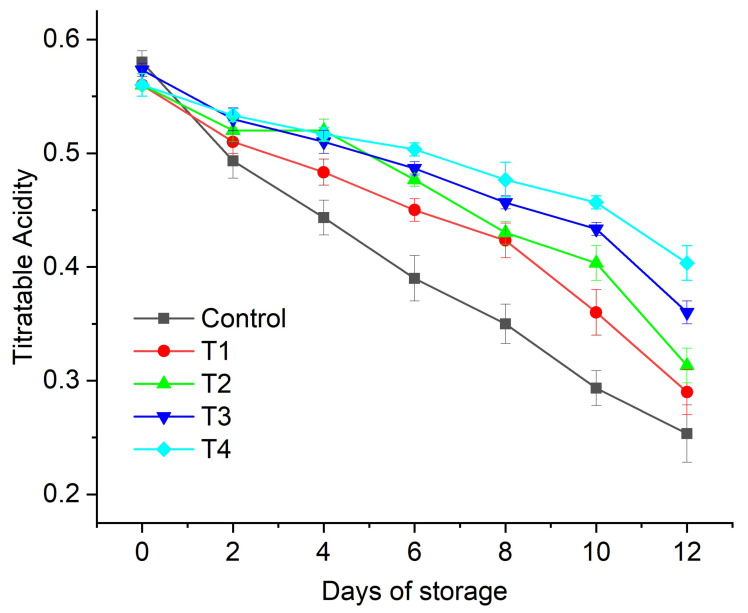
Effect of edible coatings on titratable acidity of *Ficus carica* fruit: uncoated control (water-washed), AVG (T1), AVG + glycerol (T2), AVG + glycerol + anthocyanin + 0.2% (T3), AVG + glycerol + anthocyanin + 0.5% (T4). Data are presented as mean ± SD, (*n* = 3).

The TA of the treated and untreated figs gradually reduced with increasing storage time (Figure 4). The fig fruits treated with T2 (AVG + glycerol), T3 (AVG + glycerol + anthocyanin 0.2%), and T4 (AVG + glycerol + anthocyanin 0.5%) showed a slower reduction in TA compared to the control (water-washed) and T1 (AVG)-treated fruits. Between T3 and T4, a lower reduction in TA was observed in the figs coated with T4 compared to T3. The significantly lower reduction in the coated figs could be attributed to the restriction of the respiration rate and water loss in the fruits [54]. The TSS were significantly reduced in the coated fruits compared to the uncoated control fruits after 10 days of storage due to the increase in the respiration rate and fruit maturity in the uncoated fruits compared to the coated fruits. Based on our findings, we postulated that the anthocyanin-containing coatings (T3 and T4) decreased the oxidation and fruit cellular senescence, resulting in a higher TA compared to the other coatings over the 12-day storage period. These results supported the changes observed in the pH values, with the coated samples showing significantly lower pH changes over the storage period compared to the uncoated (T0) fruits.

#### 2.3.4. Total Soluble Solids (TSS)

**Figure 5:**
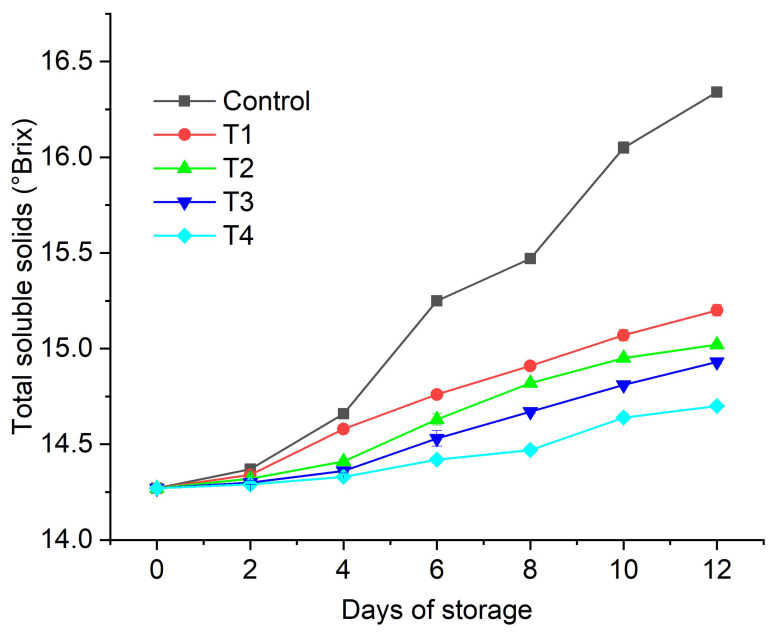
Effect of edible coatings on total soluble solids of *Ficus carica* fruits: uncoated control, AVG (T1), AVG + glycerol (T2), AVG + glycerol + anthocyanin 0.2% (T3), AVG + glycerol + anthocyanin 0.5% (T4). Data are presented as mean ± SD, (*n* = 3) (error bars are significantly smaller than the data points).

The TSS are a very important characteristic of fruits and vegetables, being indicative of their freshness and sweetness. We observed an increase in the TSS of all the treated and control fruits with increasing storage time (Figure 5). However, we observed a marginal increase in the coated figs compared to the uncoated control fruits, which exhibited the highest TSS value (16.34 ± 0.02 °Brix at day 12). Furthermore, the lowest increase in TSS was observed in the figs coated with T3 (AVG + glycerol + anthocyanin 0.2%) and T4 (AVG + glycerol + anthocyanin 0.5%). The superior performance of the T3 and T4 coatings could be ascribed to the reduction in water loss and the minimized oxidation of the fruits due to the presence of anthocyanin [21]. On the 12th day of storage, the higher TSS (14.93 ± 0.01 °Brix) value was observed for the fruits coated with T3 compared to the T4-coated fruits (14.70 ± 0.01 °Brix). Similar trends in results for fig fruits have been reported previously [55]. In study [55], a chitosan and alginate emulsion coating enriched with olive-oil was shown to inhibit a TSS increase in coated figs. The presence of anthocyanins as an active agent in an AVG-based coating has been previously shown to maintain the TSS of fruits throughout the storage period due to natural fruit ripening processes [56,57]. Furthermore, an increase in TSS during storage could be linked to the transformation of pectic compounds, starch hydrolysis, and the solubilization of polyuronides and hemicelluloses in fruit cell walls, as well as the hydrolysis of insoluble polysaccharides into simple sugars [58,59]. The incorporation of polysaccharides to support bioactive compounds from plant sources could be a potential way to extend the shelf life of fresh fruit during postharvest storage [47]. In addition, the performance of our AVG + glycerol + anthocyanin coatings in maintaining fruit TSS was similar to that of other types of coatings reported previously, including a alginate + black cumin extract coating on guava fruit [44], a sodium alginate + cinnamaldehyde-loaded nanostructured lipid carrier coating on date palm fruit [60], and a chitosan coating on sweet cherry cultivars [61]. Our AVG + glycerol + anthocyanin coatings performed better in maintaining TSS levels over the storage period than other coatings reported in the literature, including a chitosan + quinoa protein + thymol nanoemulsion coating on refrigerated strawberries [48] and a starch + mango peel powder coating on apple slices [62].

## 3. Conclusions

Edible coatings continue to attract significant attention as means to extend the shelf life of perishable foods. In this study, we explored the potential of aloe-vera-based edible coatings. *Aloe vera* gel (AVG) enriched with anthocyanin was developed as an edible coating to improve the shelf and storage life of fig fruits. Different edible coatings were tested on fig fruits over a 12-day storage period under regular refrigerated conditions. We observed significant improvements in coating performance and the maintenance of fruit quality with the inclusion of anthocyanin in the AVG coatings, as judged by the weight of the coated fruits and the changes in their pH, total soluble solids, and titratable acidity. The AVG + anthocyanin (0.5%) (T4) coating extended the fruit shelf life by limiting weight loss (~4%) compared to the uncoated control fruit (which lost >10% weight) after 12 days of storage at 4 °C, due to the loss of water from the fruits. The T4 coating also preserved fruit acidity (20% reduction in acidity) and total soluble solids (0.25 °Brix) compared to the uncoated control fruits (with a ~60% reduction in acidity and 2.5 °Brix total soluble solids). The coating performance improved significantly with an increasing amount of anthocyanin, with the T4 coatings exhibiting better performance in preserving the innate properties of coated fig fruits compared to the T3 (AVG + anthocyanin (0.2%)) and neat AVG (T1) coatings, as judged by the changes in weight loss, titratable acidity, and total soluble solids. Overall, this study found that the inclusion of anthocyanin in natural edible nanocomposite coatings can significantly improve the coating performance and shelf life of coated fruits. In the future, we hope to carry out a taste test of fruits coated using AVG + anthocyanin coatings. The developed strategy and coatings are envisaged to inspire further research exploring the commercial use of naturally occurring anthocyanin in edible coatings.

## 4. Material and Methods

### 4.1. Materials

Fresh and homogeneously sized figs (*Ficus carica*), free from physical and microbial damage, were procured from a local market in Basrah, Iraq in July 2020. The fruits were chosen based on their size, maturity stage, color, and the absence of visible defects, and they were transported in refrigerated containers to the laboratories of the Department of Food Sciences of the College of Agriculture at the University of Basrah. Fresh *Aloe vera* (*Aloe barbadensis* Miller) leaves were collected from a local orchard in Basrah city, Iraq.

### 4.2. Experimental Methods

#### Preparation of *Aloe vera* Gel (AVG)

For the preparation of AVG, the leaves were washed using chlorinated water and dried to remove dirt and contamination. The margin of the leaves was mechanically cut to remove the external epidermis and extract the gel. We then added 1% (*w*/*w*) vitamin C to the obtained gel and stirred for 30 min at 50 °C to prevent browning, and the mixture was stored in airtight, opaque glass containers for further analysis and use.

### 4.3. Physiochemical Analysis of AVG 

#### 4.3.1. pH

The pH of the AVG was analyzed by AOAC (2010) standard methods. The pH of the samples was assessing using a pH meter (pH-EMCO-256071, Japan). After the homogenization of the samples, pH was measured by the direct immersion of the electrode. 

#### 4.3.2. Moisture Content

AOAC (2012) gravimetric techniques were used to determine moisture content. Ten grams of each sample was weighed and dried in an oven for 24 h at 120 °C (Heraeus, Hanau, Germany). The change in the weight was determined using the standard gravimetry method and presented in terms of sample mass loss (percentage).

#### 4.3.3. Viscosity

An Ostwald viscometer size D was used to measure the viscosity of the AVG at 21 °C, which was calculated using the following equation:

$$r_{1}=\frac{r_{2}\;\times \;p_{1\;}\times \;t_{1}}{p_{2}\;\times \;t_{2}}$$

where *r*_1_ = viscosity of the gel, *p*_1_ = density of the gel, *r*_2_ = viscosity of water, *t*_1_ = gel descent time in seconds, *p*_2_ = density of water, and *t*_2_ = water descent time in seconds.

#### 4.3.4. Refractive Index

The refractive index of the AVG was measured using an Abbe Refractometer (A87117, Bellingham, UK). At 22 °C, the refractive indices ranged between 1.3000 and 1.7000, with an accuracy of 0.0003. Instrument calibration was conducted using distilled water. Data are presented as the average ± standard deviation of five measurements. 

### 4.4. Anthocyanin Pigment Extraction from Red Onion Peel

For the extraction of anthocyanin from red onion peel, 10 g sections of fresh red onion (*Allium cepa*) peel were washed with tap water to remove impurities. Clean wet peels were then dried in an oven at 50 °C for 24 h. Dried peels were then grounded to obtain a powder. To extract anthocyanin, 10 g of clean, dried onion peel powder was dispersed in 40 mL of acidified ethanol and 1% ascorbic acid, and the mixture was stirred for 1 min using a magnetic hot plate stirrer (GFL, Korea). The solution was incubated overnight at 30 °C in an oven (Binder) for the extraction and recovery of anthocyanin pigments. The obtained solution was filtered using Whatman filter paper (540), after which the solvent was evaporated using a rotary evaporator (Franklin Electric, UK) at 40 °C to obtain dry powdered anthocyanin. The obtained anthocyanin pigment powder was stored in a sterile container for further use.

### 4.5. Preparation of AVG-Based Coating 

AVG was diluted with distilled water to 40% (*v*/*v*) as a coating solution. We added 2% glycerol as a plasticizer, and the solution was homogenized using a magnetic stirrer at 1500 rpm for 6 h. Anthocyanin (0.2 mg/100 mL and 0.5 mg/100 mL) was added to the coating solution as an antioxidant agent. The pH of the coating mixture was maintained at 4 using citric acid (4.5–4.6 g/L). We placed 15 mL of each AVG solution into petri dishes to dry at 45 °C in a sterilized oven to obtain different coatings. 

### 4.6. Microstructure Analysis 

The surface morphology of the AVG-based edible coatings enriched with anthocyanin was analyzed by a scanning electron microscope (SEM) (Supra 55 VP, Carl Zeiss, Germany) with a voltage of 1 KV and a magnification power of 1000–15,000×. The results are expressed in terms of the appearance of the scattered electrons at different magnification powers.

### 4.7. Application of AVG-Based Coatings Enriched with Anthocyanin

Fig fruits was carefully washed with chlorinated water to remove any foreign debris, such as dust and dirt. Maximum effort was put into selecting fruits that were uniform in size, high in quality, and free from injury or disease. The different formulations of edible coatings, i.e., AVG (T1), AVG + glycerol (T2), AVG + glycerol + anthocyanin + 0.2% (T3), and AVG + glycerol + anthocyanin + 0.5% (T4), were applied to the fruits. The deposition of coating materials on the fruits was carried out via a dipping procedure for 5 min, and fruits were dried using an oven at 45 °C for 30 min. The fruits were stored under refrigerated conditions at 4 °C throughout the storage period of 12 days. Deionized water used as a control (T0) to treat fig fruits. The postharvest quality evaluation was performed at two-day intervals (i.e., on days 2, 4, 6, 8, and 12). 

### 4.8. Scanning Electron Microscopy (SEM) Imaging of Edible Coatings

We conducted SEM imaging of coating samples, which were prepared by dropcasting 0.5 mL of each coating solution onto a copper stub and drying under ambient conditions. Dried coatings were gold-coated prior to imaging using an SEM (Zeiss SUPRA 55VP, Germany) at a 10 kV accelerating voltage.

### 4.9. Physicochemical Analyses of Ficus carica Fruit

To prepare samples for physicochemical analysis, 10 g of uncoated and coated fruits at different time points (2, 4, 6, 8, 12 days) were homogenized with 80 mL distilled water using a kitchen blender (MX-KM5070, Panasonic, Malaysia). The obtained homogenized fruit was squeezed through a muslin cloth to obtain fruit juice, which was used for further analysis.

#### 4.9.1. Weight Loss

The weight loss of control and treated fig fruits was calculated using the mass difference technique, as reported previously [12]. The fruits were weighed every 2 days over the storage period of 12 days using an analytical weighing balance. Weight loss was calculated by taking the difference between fruit weights at specific time points and day 0. Three repeats were performed under all conditions at each time point, and data are presented as an average ± standard deviation.

#### 4.9.2. pH

The pH of the extracted fruit juice was determined using the standard AOAC (2010) method. A 10 mL sample of juice from control (water-washed fruit) and treated fig fruits was placed in a beaker, before an electrode of the digital pH meter (pH-EMCO-256071, Japan) was dipped inside the beaker containing the sample and left for 10 min. This technique was carried out a minimum of three times for all samples, and data are presented as average ± standard deviation. Before use, the pH meter was calibrated with buffer solutions of pH 4 and 7.

#### 4.9.3. Titratable Acidity (TA)

The TA of control and AVG-coated fruits was measured using the titration method reported previously, with minor modifications [32]. A 25 mL sample of extracted fruit juice prepared using 10 g of fruit pulp in 40 mL distilled water was titrated with 0.1 N NaOH solution. Phenolphthalein was used as an indicator for marking the end point. TA was measured as malic acid (%) and calculated using the equation below:

$$Titratable\;acidity\;\left(\%\right)=\;volume\;of\;NaOH\;\times \;miliequivalent\;weight\;of\;acid\;\times \;normality\;of\;NaOH\;\times \;volume\;of\;sample$$

#### 4.9.4. Total Soluble Solids (TSS)

The TSS of the control and treated fruits were estimated using a refractometer (A87117, Bellingham, UK) with a precision of ±0.1%. Distilled water was used to calibrate the refractometer. Two drops of fruit juice were placed on the refractometer prism, and the measurements were taken again. This procedure was repeated three times for each fruit sample, and the prism was cleaned with ethanol after each measurement.

## Figures and Tables

**Table 1:** Physicochemical characteristics of AVG.

Characteristic Parameter	Mean± Standard Deviation
Moisture	97.52 ± 0.83
pH	5.29 ± 0.037
Acidity	0.06 ± 0.00
Carbohydrates	0.65 ± 0.01
Viscosity	4.66 ± 0.00
Refractive index	1.33 ± 0.00
Total soluble solids	3.10 ± 0.00

## Data Availability

Data are available on request from the authors.
